# A decision support tool with health economic modelling for better management of DVT patients

**DOI:** 10.1186/s13561-022-00412-9

**Published:** 2022-12-26

**Authors:** Reda Lebcir, Usame Yakutcan, Eren Demir

**Affiliations:** University of Hertfordshire, Hatfield, AL10 9AB UK

**Keywords:** Deep vein thrombosis, Community services, Discrete event simulation, STAR, Decision support tool, Patient flow modelling, National health service, United Kingdom

## Abstract

**Background:**

Responding to the increasing demand for Deep Vein Thrombosis (DVT) treatment in the United Kingdom (UK) at times of limited budgets and resources is a great challenge for decision-makers. Therefore, there is a need to find innovative policies, which improve operational efficiency and achieve the best value for money for patients. This study aims to develop a Decision Support Tool (DST) that assesses the impact of implementing new DVT patients’ management and care policies aiming at improving efficiency, reducing costs, and enhancing value for money.

**Methods:**

With the involvement of stakeholders from a number of DVT services in the UK, we developed a DST combining discrete event simulation (DES) for DVT pathways and the Socio Technical Allocation of Resources (STAR) approach, an agile health economics technique. The model was inputted with data from the literature, local datasets from DVT services, and interviews conducted with DVT specialists. The tool was validated and verified by various stakeholders and two policies, namely shifting more patients to community services (CSs) and increasing the usage of the Novel Oral Anticoagulant (NOAC) drug were selected for testing on the model.

**Results:**

Sixteen possible scenarios were run on the model for a period of 5 years and generated treatment activity, human resources, costing, and value for money outputs. The results indicated that hospital visits can be reduced by up to 50%. Human resources’ usage can be greatly lowered driven mainly by offering NOAC treatment to more patients. Also, combining both policies can lead to cost savings of up to 50%. The STAR method, which considers both service and patient perspectives, produced findings that implementing both policies provide a significantly higher value for money compared to the situation when neither is applied.

**Conclusions:**

The combination of DES and STAR can help decision-makers determine the interventions that have the highest benefits from service providers' and patients’ perspectives. This is important given the mismatch between care demand and resources and the resulting need for improving operational and economic outcomes. The DST tool has the potential to inform policymaking in DVT services in the UK to improve performance.

**Supplementary Information:**

The online version contains supplementary material available at 10.1186/s13561-022-00412-9.

## Introduction

Demand for healthcare services has increased dramatically all over the world driven by ageing populations, changing lifestyles, higher incidence and prevalence of chronic diseases, frequent occurrence of pandemics, and poor social and economic conditions [1]. This created the need for deploying more resources to cope with demand inflating, as a result, the costs of delivering healthcare [2]. Parallel to this, there have been extended periods of fiscal austerity and cuts to healthcare budgets, which created significant challenges to decision-makers on how to best allocate scarce resources to deal with increased demand and deliver effective and high-quality care to patients [3].

The United Kingdom (UK) is not an exception to this situation as the country witnessed a steady increase in healthcare demand in the last two decades. During the same period, fiscal austerity following the financial crisis in 2008 led to tighter budget allocations to the National Health Service (NHS), the publicly funded organisation responsible for delivering healthcare in the UK free of charge for patients. This combination of high demand and reduced budgets created a shift in management paradigms in the NHS. Decision-makers need to deliver healthcare to patients in new and innovative ways which are: (i) viable and sustainable from an economic and cost perspectives, and (ii) do not have a negative impact on patients’ clinical outcomes and quality of care [4].

The management and treatment of patients with Deep Vein Thrombosis (DVT) illustrate the importance of pursuing innovative care delivery methods to achieve better clinical and cost outcomes. DVT is a blood clot, which forms within the deep veins generally in the legs, but also in veins of the arms, and the mesenteric and cerebral veins. It is a common and serious disease, which accounts for most cases of pulmonary embolism and it is the third most common cause of death from cardiovascular disease following heart attacks and strokes [5]. The risk factors associated with DVT include obesity, immobility, smoking, age, presence of cancer and heart disease, and gender [6]. The annual number of DVT-related deaths within 90 days following hospital discharge increased by 3%, from 8,025 in 2007/08 to 8,301 in 2018/19 [7]. The cost of treating DVT patients reached approximately £159 Million in 2017/18 [8]. This increasing demand for DVT treatment and associated costs warrant changes to the current care delivery processes to improve efficiency and value for money.

Two recent promising developments have taken place, which could improve the clinical outcomes of DVT treatment and reduce the costs of providing care to patients. From a treatment perspective, a new class of drugs known as Novel Oral Anticoagulant (NOAC) are now available and are associated with better clinical outcomes than the current standard treatment (e.g., Warfarin). From a care delivery perspective, it is becoming possible to deliver DVT treatment in Community Services (CSs) at a fraction of the costs in hospitals and there is established evidence that delivering treatment in CSs reduces costs and enhances patients’ satisfaction with quality of care [3].

The introduction of the above changes in existing DVT care services requires a careful analysis to determine whether clinical and cost improvements are achievable and, if so, what is the magnitude of the improvement. The reason is that health systems are complex, and it is not always guaranteed that a change to some of their elements will lead to a better performance of the whole system. The reason is that health systems include interconnected elements and making changes to one element without taking into account the ramifications on the other ones may defeat the aim of the change. For example, improving the screening technology for cancer may help identifying more cases and, consequently, increase the demand for cancer treatment. However, if there are no sufficient treatment resources and capacity, this would simply increase the backlog of cases waiting for treatment, more complications for patients as they wait for longer periods to access treatment, and increased death rates. Responding to this by putting more pressure on staff to treat more patients, as it is widely the case in health services, will only lead to more staff fatigue, burnout, sickness leave, and lower quality of care, compounding the problem. Given the above-cited challenges faced by the NHS DVT services, the primary aim is to determine whether the new changes (introduction of the NOAC treatment and shifting patients from hospitals to CSs) will enable these services to cope with higher demand, reduced costs, and improve efficiency, that is the ability to treat more patients with decreasing level of treatment resources and within allocated budgets. Determining this in advance of implementing any changes in the real world is critical to provide local NHS DVT services managers and commissioners, and Department of Health officials with evidence that the innovative policies will lead to the expected improvement so that they can proceed with the implementation of the changes with confidence.

Simulation methodologies have been applied extensively in the healthcare sector to provide such evidence and estimate the scale of possible improvements, which can be achieved if new policies and processes are implemented. There are a number of simulation methodologies, which have been applied in healthcare including (i) Discrete Event Simulation (DES), (ii) System Dynamics (SD), and (iii) Agent Based Simulation (ABS). DES is generally used to represent the operational processes where uncertainty and resource constraints are important features of the represented context, and where health economic (cost-effectiveness) considerations can also be of interest. SD is applied mainly to represent dynamically complex healthcare systems when there are dense and circular (feedback) relationships between the system’s elements. ABS focuses on the behaviour of agents (e.g., patients) in a healthcare system and how behaviour changes, due to interactions between the agents, affect the overall system. DES, which can be applied in different settings, mimics processes and patient clinical history at a discrete set of points in time. Given that the DVT care system involves many uncertainties (e.g., level of demand, disease progression, treatment outcomes) and requires resources (e.g., nurses, doctors), DES is the selected simulation methodology for this study.

In addition to establishing whether changes can lead to efficiency improvements, decision-makers, especially services commissioners, are interested in determining if the changes also offer good value for money. The real-world implementation of new processes requires investments and resources, and it is becoming more important to evaluate if these investments are worthwhile and justify the allocation of, what is in the case of the NHS, scarce financial resources.

Cost-Effectiveness Analysis (CEA), which aims to inform decisions regarding the allocation of scarce resources, has been used extensively to determine the changes and interventions, which are worth investing in [9]. CEA includes a wide umbrella of methodologies and approaches, which have been used to achieve its aim. For example, CEA has been extensively used by the UK National Institute for Clinical Excellence (NICE) to recommend which drugs should be funded by the NHS [10]. However, the CEA methodology, as applied by NICE, is suitable for single “accept-reject” decisions and does not account for variations in local stakeholders’ preferences, how they rank and value different interventions, and the softer subtle criteria they use to evaluate interventions [11]. It is, therefore, not suitable to guide local decisions regarding the allocation of a mix of resources for the services to be provided, where trade-offs need to be made to allocate a fixed budget to implement policies [12].

Socio-Technical Allocation of Resources (STAR) is another CEA approach, which is more appropriate for use in local contexts to inform decisions regarding the allocation of resources within a budget from current to new more beneficial interventions [11]. STAR gives decision-makers a tool to conduct economic evaluations in the real world by engaging directly with staff involved in the health context and taking account of their views when evaluating different interventions. Therefore, STAR is an appropriate approach to evaluate the value for money of the changes to the DVT care pathways cited above where “use of NOAC” and “shifting patients from hospitals to CSs” can be conceptualised as the new groups of beneficial policies to be evaluated for allocation of resources.

The aim of this paper is, therefore, to evaluate the impact of implementing these policies on the DVT care pathways’ efficiency treatment cost, and interventions’ value for money. The combination of DES and STAR in this evaluation broadens the scope of analysis beyond the traditional focus on efficiency and cost reduction to include health economics considerations. As such, the evaluation is more comprehensive as it covers both the operational and economic aspects and will provide both DVT services managers and commissioners with the evidence to determine the changes and interventions most beneficial for implementation.

## Methods

### The rationale for selecting DES

DES is a recognised methodology for its suitability and adoptability in analysing healthcare contexts, representing their structural complexities, and providing robust evidence to improve their performance [13, 14]. In the current research, DES is appropriate because of its ability to represent the structure of the care pathways and the time-related evolution of patients over these pathways. Patients are conceptualised as entities and as time unfolds, their transitions on the different parts of the pathways are tracked in the model [15]. In addition, DES enables the representation of uncertainties, which are common features of healthcare delivery (for example, the possibility that a patient will develop DVT following surgery). Individual characteristics such as age, gender, and co-morbidities, which influence the patients’ “route” on the care pathway and treatment outcome can also be included in a DES model.

Also, the DES` “What-if” facility enables testing of alternative scenarios reflecting new policies and interventions (for example introducing a new drug to treat DVT). These scenarios can be run on the DES model to predict the magnitude of possible performance improvement should the policies and interventions get implemented. This gives policymakers information and evidence on the changes to implement and improves the policymaking process. DES has been successfully applied in healthcare contexts for more than 40 years [16, 17], e.g., for capacity planning [18], emergency departments [19], chronic diseases [20, 21], infectious diseases [22, 23], and the use of community services for patients with Parkinson’s disease [3].

### DES applications to health economics

Although DES is more commonly applied in the contexts of demand, resource, and capacity, the use of DES for economic analysis (i.e., the cost-effectiveness of interventions) has become popular too. There is increased acceptability that it is an adequate modelling methodology for this analysis as evidenced by recently published research [24]. For example, a CEA focusing on the impact of an assisted pharmacotherapy-based smoking cessation intervention compared to unassisted ones was conducted via a DES model [25]. In another study, Hartz et al. [26] used a DES model to perform a CEA of two interventions for the treatment of Alzheimer's disease patients with a specific drug. Jahn et al. [27] compared the cost-effectiveness of two different treatments of Coronary Artery Disease, taking into account capacity constraints and dynamic waiting lists represented through a DES model. Another example is that a CEA was performed using a DES model for Multiple Myeloma patients to compare a combination of two different drugs against a single one to treat patients [28]. Other similar applications focused on the surgical pathways of Colorectal cancer [29] and laparoscopy [30]. Lastly, DES was used for CEA of evaluating organisational changes to healthcare services. For example, Rejeb et al. [31] assessed different configurations of Health Information Systems (HIS) in an Oncology service to determine the best one for implementation.

## Model development

### Model interface

**Fig. 1:**
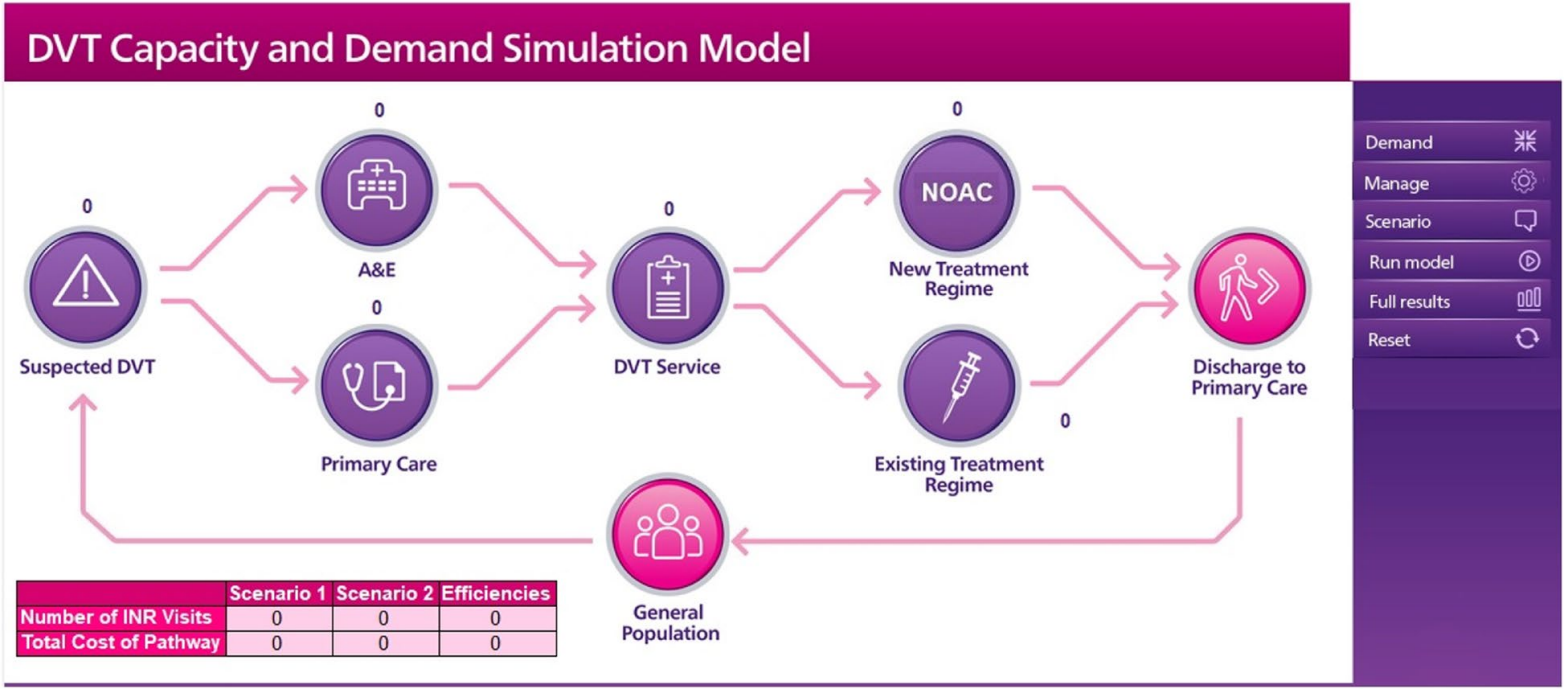
Interface of the DVT patients’ pathways DES model

We developed a DES simulation model representing the pathways of DVT patients covering admission, treatment, and discharge processes. The model structure represents the complexity of the pathways and the journey of patients over them. However, given that the primary interest of decision-makers is the use of the model to inform policymaking rather than understanding its technical complexity, the DES model was converted into a Decision Support Tool (DST) by integrating the DES model with a front user-friendly interface (Fig. 1). The DST is designed to be used by key decision-makers in mind, including service managers, senior nurses, and physicians without the need for technical knowledge in DES. As a result, the front interface and the simulation controls are simple, concise, and fit for purpose. Users can make necessary changes in the input parameters. The input parameters are in an Excel spreadsheet for ease of use. The user of the model can customize the input values for many parameters including demand, costing, treatment, and patient routing. In this sense, the model is generic and can be tailored for specific healthcare providers. A training session in the form of a series of workshops for key decision-makers was provided by the development team.

The DST includes two configurations with regard to the treatment of DVT patients. The first configuration represents the situation whereby the existing treatment regime is applied, and the second configuration represents the situation whereby the new treatment regime, which includes “use of NOAC” and “shifting patients from hospitals to CSs”, is applied. The DST users can choose the number of years the simulation model will run and after the simulation run, summary output metrics including, for example, the number of anticoagulation (known also as INR) clinic visits and total cost are shown on the interface. In addition, an exhaustive set of outputs are available to decision-makers in the form of an Excel spreadsheet with numerical and graphical outputs (including STAR/value for money calculations).

### Description of the DES model

**Fig. 2:**
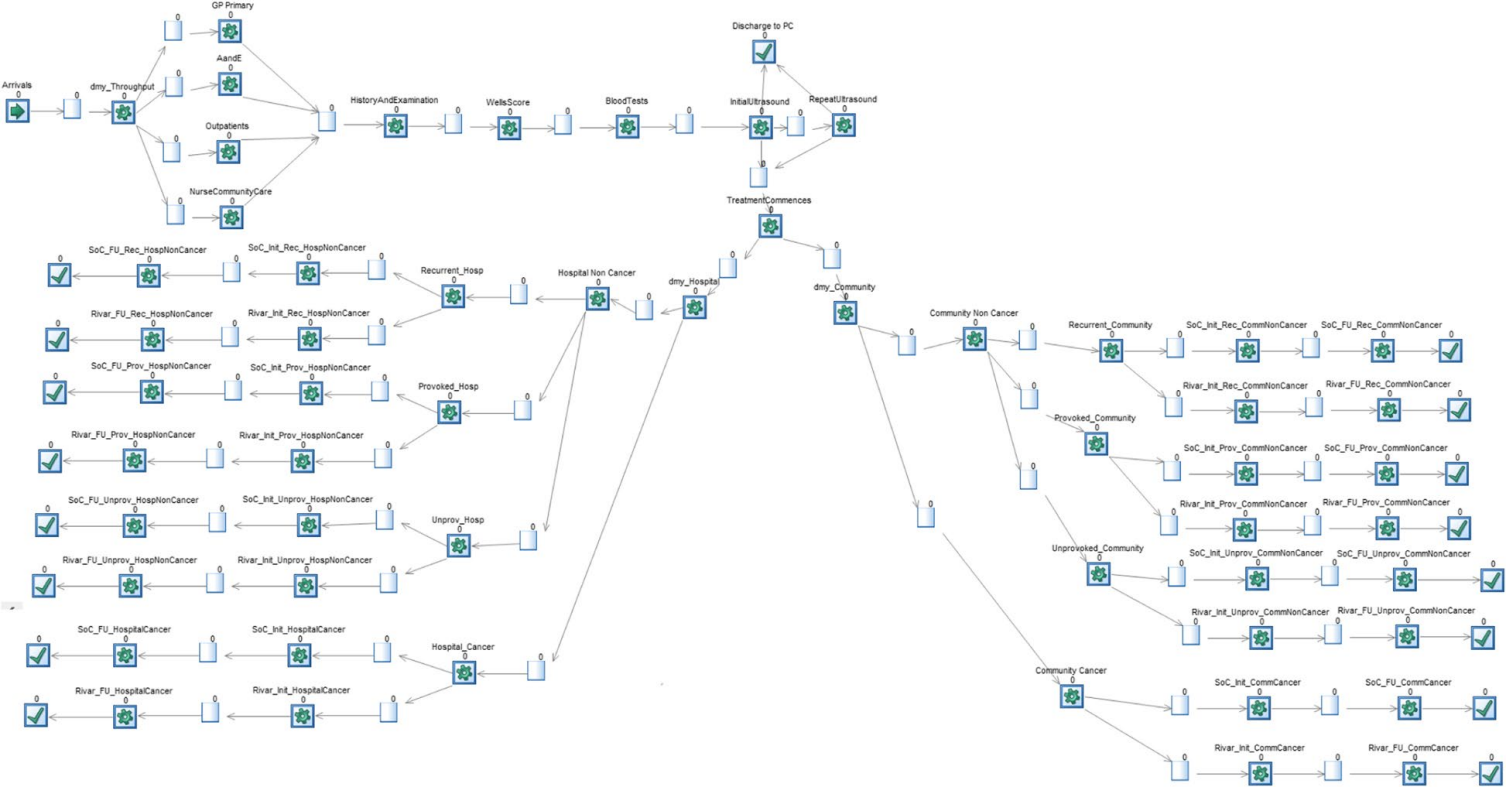
Structure of the DVT patients’ pathways DES model

The detailed patient flow diagram is shown in Fig. 2 with Simul8 icons, the simulation software used for modelling. Patients arrive in the hospital for DVT services following a referral from a primary care GP, accident and emergency unit, outpatient clinic, or community care service. Following referral, the patient goes through a sequence of diagnostic procedures including evaluation of patient history, examination by a consultant, assignment of Wells Score, blood test (D-dimer test), and ultrasound. Depending on these tests’ results, a decision is made on whether the patient is discharged if the tests indicate no presence of DVT or put on a treatment phase if DVT is detected. The treatment phase involves a number of visits to a specialised hospital with INR clinics and patients are discharged following the conclusion of the treatment phase.

The model also represents the different groups of patients, which are referred to DVT treatment services. The first group is called “provoked” and includes patients who are known to be at risk of developing DVT such as pregnant women, those undertaking surgery, or suffering from trauma. The next group is called “unprovoked” and includes patients, who develop DVT without a clear clinical reason. The last group called “recurrent” includes the patients who had DVT before and developed it another time.

Patients under the standard treatment require on average 9, 14, and 24 INR visits over 3, 6, and 12 months respectively, following the start of the treatment phase. These patients are treated with Low Weight Molecular Heparin (LWMH) during the first 8 days of the treatment phase followed by Warfarin for the remaining treatment duration. On the other hand, those on the NOAC treatment require two INR visits over the whole treatment phase whether this lasts for 3, 6, or 12 months and involves NOAC tablets. The high number of visits under the standard treatment is due to the side effects of Warfarin, which requires frequent checks to avoid any complications to the patient. All these treatment regimens are from clinical guideline and implemented by the NHS for the treatment of DVT.

The progression of patients through the pathway shown in Fig. 2 is subject to resources’ availability and constraints as different testing and treatment activities require a mix of resources to take place. For example, a patient treated at the hospital needs a bed and a consultant whereas treatment at community services can only take place if a nurse is available. In line with these resource requirements, patients may need to wait for the start of treatment until the corresponding resources are available.

### Data sources

Most of the input parameters are pre-determined through an in-depth review of the literature and, on a small number of occasions, healthcare professionals (i.e., nurses and consultants) have provided their expert opinion, e.g., time to treatment. We also utilised publicly available data on NHS Digital and NHS England websites, covering many aspects of health and social care services, e.g., hospital activity, reference cost, national tariffs, and waiting times [32, 33].

A participative modelling (PM) approach with key stakeholders was used to support the modelling process, which was also useful for collating the relevant information for developing the model. The PM approach enabled stakeholders to share their views in a safe environment about the existing DVT pathway (e.g., diagnosis, treatment, monitoring, patient outcomes), whilst also evaluating proposed scenarios for improvement purposes. A final workshop with participants was provided with a summary of the project and the updated model presented.

### Parametrisation and validation of the model

Following the development of the simulation model on SIMUL8, a list with the input data required to parametrise it was derived. The input data is divided into categories related to costs, resources, treatment, and value for money. Costs data cover the cost of first and follow-up visits to hospital and community service including per day and per tablet/injection cost. Resources data include the level of human resources available including haematologists, nurses, and radiologists, and the fraction of time these resources allocated to treat DVT patients. Treatment-related data focus on the percentage of DVT patients who are treated in hospital or in community services, and the expected treatment durations broken down by patients’ groups (provoked, unprovoked, and recurrent cases). Value for money data includes the pathways costs for the interventions, population health benefits, and feasibility of the health benefits. In addition, the model incorporates a total budget and quantitative scores for benchmarking for each type of intervention. The total data list covered 70 input parameters, and these were derived and extracted from the literature, local datasets from collaborators, and the interviews conducted with DVT specialists from a number of hospitals in the UK. The list of input parameters is given in the supplementary file (See Additional file 1: Table S1).

The model was subjected to verification and validation tests to ensure its robustness and ability to generate results close to those observed in the real world. The model structure (i.e., the patient pathway) was verified (prior to development in a simulation environment) by the stakeholders involved in the running of DVT services in many hospitals in the UK (University Hospitals NHS Trust, Bristol Royal Infirmary, Royal Liverpool University Hospital, Royal Eye Infirmary, Broomfield Hospital, and Lansdowne Hospital). This process took place throughout the model building cycle. The stakeholders’ feedback was used to refine the model until they were satisfied that it reflects the actual DVT care system.

The DVT simulation model was validated to ensure it is suitable for further experimentation with other scenarios. Validation tests were conducted by comparing the model outputs (e.g., the number of clinic visits) against the real-world data. The difference between the simulation results and observed data was within 5% either side of the observed data. Thus, the test results indicate that the model is deemed valid and ready to be used for scenario runs. The model`s code is not publicly available due to its complexity and potential intellectual property implications. However, the intensive validation of the model enabled us to confirm its accuracy and reliability for use in practice, just like with most simulation models.

## Simulation scenarios and results

### Selection of scenarios

As discussed earlier, the aim of this study is to evaluate the impact of new policy interventions, namely the “use of the NOAC treatment” and “increased use of community services” to treat patients, on a number of performance indicators to determine which interventions have the highest benefit for patients and provide the best value for money for the service provider (in this case the NHS).

The process of selecting the possible scenarios for simulation on the model involved extensive discussions with the DVT physicians and nurses, who informed the development and validation of the model. Following these discussions, 16 scenarios were identified, each combining one out of 4 possible increases in the use of community services (Low, Medium, High, Very High) and one of 4 possible increases in the level of usage of the new NOAC treatment (Low, Medium, High, Very High). Discussions also indicated that low, medium, high, and very high correspond to a 10%, 20%, 40%, and 50% increase respectively. The list of scenarios is presented in Table 1. It is important to note that the initial scenario named “Baseline Scenario” is the standard of care, where all patients are treated in hospital with the Warfarin drug, that is no patient is treated with NOAC (0% NOAC) and in CSs (0% CSs).

**Table 1:** The list of scenarios

Scenario	% of patients treated in CSs	% of patients treated with NOAC
Baseline	0%	0%
Scenario 1	10%	10%
Scenario 2	10%	20%
Scenario 3	10%	40%
Scenario 4	10%	50%
Scenario 5	20%	10%
Scenario 6	20%	20%
Scenario 7	20%	40%
Scenario 8	20%	50%
Scenario 9	40%	10%
Scenario 10	40%	20%
Scenario 11	40%	40%
Scenario 12	40%	50%
Scenario 13	50%	10%
Scenario 14	50%	20%
Scenario 15	50%	40%
Scenario 16	50%	50%

*CSs* Community Services, *NOAC* Novel Oral Anticoagulant treatment

Following the selection of scenarios, a further discussion took place with the participants to identify the most appropriate performance indicators to evaluate the scenarios given that the aim of the paper, as stated above, is to evaluate the impact of implementing the policies on the DVT care pathways’ efficiency, treatment cost, and interventions’ value for money. Efficiency is represented by the performance indicators: “treatment activity”, and “human resources’ utilisation”. Treatment cost is represented by the indicator “total costs”, and interventions’ value for money is represented by the indicator “Value for Money score” (calculated through the STAR approach). Definitions of the performance indicators are given in Table 2.

**Table 2:** Definition of the performance indicators

Performance Indicator	Definition
*Operational Outcomes*	
Total Number of INR visits	Total number of visits by DVT patients in anticoagulation clinic (including first and follow-up visits)
Total Nurse service hours	Total number of service hours provided by nurses for the treatment of DVT patients
Total Doctor service hours	Total number of service hours provided by doctors (i.e., haematologists) for the treatment of DVT patients
*Financial Outcomes*	
Cost of standard care	Cost of the current standard treatment (including drug costs, first and follow-up visits)
Cost of NOAC	Cost of new treatment with NOAC (including drug costs, first and follow-up visits)
Staff cost	Total staff costs required to treat DVT patients
Total cost	Total cost incurred to treat DVT patients, i.e., the sum of cost of standard of care, cost of NOAC, and staff cost
*Cost-effectiveness Outcomes*	
Number of patients	The number of patients who benefit from intervention
Expected Health Benefit	Total expected health benefit gained by the patients who received intervention
Value for Money	The ratio of expected health benefit from the intervention to the associated expenditure with treatment (see Appendix 1)
Total cost	Total cost associated with the treatment provided

*INR* Anticoagulation clinic, *DVT* Deep Vein Thrombosis, *NOAC* Novel Oral Anticoagulant treatment

### Simulation results

The initial size of the population included in the model was 220,000, which is a typical size of a clinical commissioning group in England [34]. The simulation model was run for a period of 5 years with a projected 1% annual increase in the number of patients leading to a total of 247 DVT patients by the end of year 5 in line with the UK DVT prevalence of 104.6 per 100,000 individuals [35]. The time horizon (i.e., 5 years) was suggested by key stakeholders to measure the short- and medium-term consequences of change. However, it is adjustable for a shorter or longer period for future use.

Before describing the simulation results, it is important to indicate that, under the baseline scenario, the number of INR visits is 60,463 requiring 9504 nurse hours and 4199 doctor hours, with a total cost of £4,585,941. These results will enable an appreciation of the possible improvements associated with the policy interventions and represented in the different scenarios. The results are described in the following for the four areas cited above.

#### (i) Treatment activity 

**Fig. 3:**
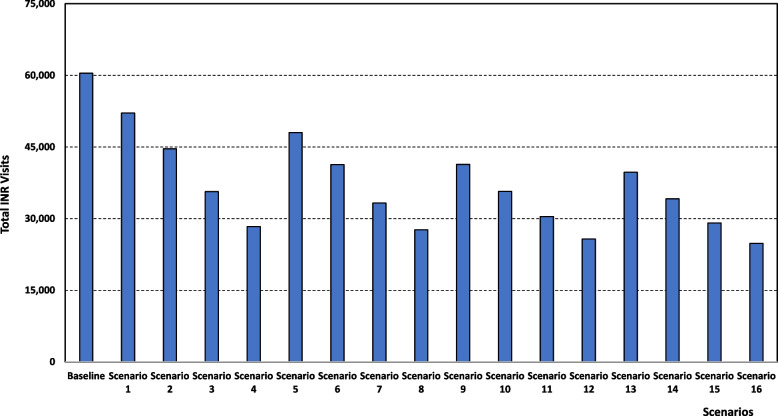
Total Number of INR visits for each scenario. *INR* Anticoagulation clinic

The policies of shifting patients from hospital to community services and treating patients with NOAC has a positive impact on the number of INR visits to the hospital both when each intervention is implemented individually, and if both are implemented simultaneously (See Table 3 and Fig. 3). The number of INR visits decreases if the fraction of patients treated with NOAC increases and this is the case for all options regarding the fraction of patients shifted to community services. As an illustration, under the situation whereby 20% of patients are shifted to community services, the number of INR visits goes down from 48,041 if 10% of patients receive the NOAC treatment (scenario 5) to 41,312, 33,268, and 27,644 if 20% (scenario 6), 40% (scenario 7), and 50% (scenario 8) of patients, respectively, are shifted with the latter scenario (scenario 8) consisting of a 54% reduction (60,463 to 27,644 INR visits) in comparison to the baseline scenario.

**Table 3:** Operational performance simulation results

Scenario	Total number of INR visits (first and follow-ups)	Total nurse service hours	Total doctor (haematologist) service hours
Baseline	60,463	9,504	4,199
Scenario 1	52,112	8,618	3,819
Scenario 2	44,615	7,537	3,356
Scenario 3	35,644	5,889	2,650
Scenario 4	28,330	5,041	2,286
Scenario 5	48,041	8,465	3,754
Scenario 6	41,312	7,446	3,317
Scenario 7	33,268	5,941	2,672
Scenario 8	27,644	5,021	2,278
Scenario 9	41,376	8,050	3,576
Scenario 10	35,694	7,154	3,192
Scenario 11	30,423	5,961	2,681
Scenario 12	25,754	5,106	2,314
Scenario 13	39,711	7,944	3,530
Scenario 14	34,140	6,999	3,126
Scenario 15	29,114	5,884	2,648
Scenario 16	24,793	5,008	2,272

*INR* Anticoagulation clinic

A similar decline trend is observed if more patients are shifted to community service (regardless of the percentage of patients under the NOAC treatment). For example, if 40% of patients are offered NOAC treatment, the number of INR visits decreases from 35,644 if 10% of patients are shifted to community services (scenario 3) to 33,268, 30,423, and 29,114 under 20% (scenario 7), 40% (scenario 11), and 50% community services shift (scenario 15), respectively, with the latter (scenario 11) corresponding to a 52% reduction compared to the baseline scenario.

If we analyse the combined effect of the two policies, we can observe that the rate of decline is sharp as more patients are moved to community services and put on NOAC treatment regime. Starting from a number of INR visits of 52,112 if both interventions are applied to 10% of patients (scenario 1), there is an increasing steep decline to 41,312, 30,423, and 24,793 if the interventions are applied to 20% (scenario 6), 40% (scenario 11), and 50% (scenario 16), respectively. The latter scenario constitutes a significant 59% gain compared to the baseline one.

#### (ii) Human resources’ utilisation 

**Fig. 4:**
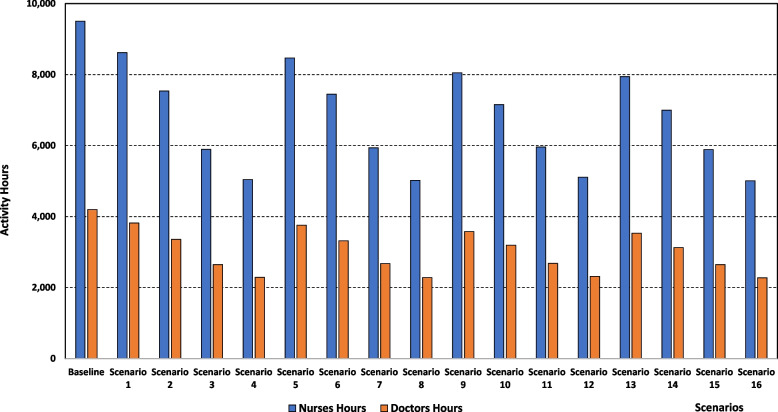
Activity hours spent by Nurses and Doctors for each scenario

The level of human resources usage, expressed in the total number of nurses’ and doctors’ hours (in hospital and community services) required to treat DVT patients, presented in Table 3 and Fig. 4, is reduced by putting more patients under the NOAC treatment and this is the case for all community services shift situations. As an example, if 50% of patients are moved to community services, the number of nurses’ hours declines from 7,944 if 10% of patients are treated with NOAC (scenario 13) to 6,999, 5,884, and 5,008 if 20% (scenarios 14), 40% (scenario 15), and 50% (scenario 16) of patients are taking NOAC, respectively, with the latter constituting a 48% (from 9,504 to 5,008) saving compared to the baseline scenario. Similarly, the number of doctors’ hours decreases from 3,530 under 10% NOAC treatment (scenario 13) to 3,126, 2,648, and 2,272 under 20% (scenario 14), 40% (scenario 15), and 50% (scenario 16) NOAC treatment, respectively. Therefore, under scenario 16, hospital doctor hours requirements are 46% less compared to the baseline figure of 4,199 h.

A similar decrease trend in human resources requirements is observed if both policies are concurrently applied. Nurses’ hours go down from 8,618 under scenario 1 (10% combined change) to 7,446, 5,961, and 5,008 under scenario 6 (20% combined change), scenario 11 (40% combined change), and scenario 16 (50% combined change), respectively, with the latter scenario requiring 48% fewer nurses’ hours than the baseline one. Doctors’ hours decline from 3,819 (scenario 1) to 3,317, 2,681, and 2,272 under scenarios 6, 11, and 16, respectively. Scenario 16 requires 46% fewer doctors’ hours than the baseline scenario.

It is important to note that human resources’ usage is not affected greatly by moving more patients to community services (independently of the faction of patients given NOAC treatment). To illustrate this, if 20% of patients are given the NOAC treatment, nurses’ hours change from 7,537 if 10% of patients are shifted to community services (scenario 2) to 7,446, 7,154, and 6,999 if the shift is 20% (scenario 6), 40% (scenario 10), and 50% (scenario 14), respectively. Regarding doctors’ hours, they are 3,356, 3,317, 3,192, and 3,126 under scenarios 2, 6, 10, and 14, respectively.

#### (iii) Total costs

**Fig. 5:**
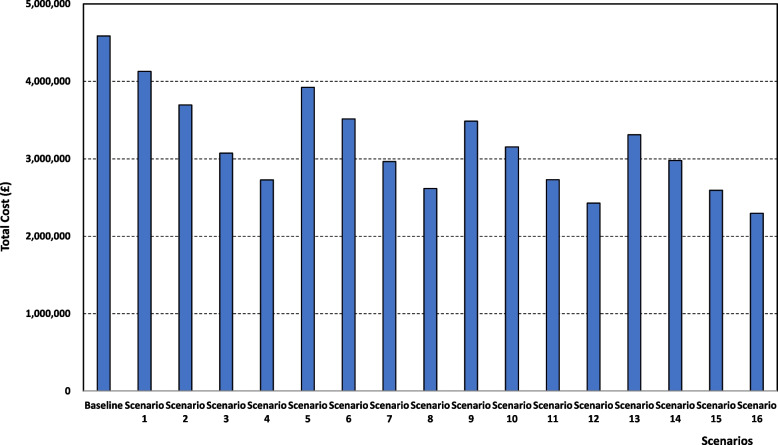
Total Cost for each scenario

The total cost, which includes the costs for standard care, NOAC care, and staff costs (See Table 4 and Fig. 5) is positively affected by putting more patients under the NOAC treatment, and this is the case for all fractions of patients shifted to community services. For example, if 40% of patients are cared for in community services, then the total cost drops from £3,484,551 under scenario 9 (10% NOAC) to £3,153,067, and £2,730,103, and £2,428,574 under scenario 10 (20% NOAC), scenario 11 (40% NOAC), and scenario 12 (50% NOAC), respectively, the latter (scenario 12) being almost half the baseline scenario total cost. These reductions are achieved even though the NOAC treatment cost is higher under the scenarios associated with increased NOAC fractions (£438,128 for scenario 12 against £143,566 for scenario 9) as this additional cost is well offset by the gains made in both the standard care cost and the staff cost.

**Table 4:** Financial performance simulation results

Scenario	Cost of standard care (incl. drug costs, FU’s and first visits)	Cost of NOAC (incl. drug costs, FU’s and first visits)	Staff cost	Total cost
Baseline	£2,811,048	£0	£1,774,893	£4,585,941
Scenario 1	£2,398,573	£116,898	£1,613,095	£4,128,567
Scenario 2	£2,054,464	£224,410	£1,415,775	£3,694,649
Scenario 3	£1,553,305	£403,630	£1,114,937	£3,071,872
Scenario 4	£1,262,138	£505,103	£960,037	£2,727,278
Scenario 5	£2,212,853	£125,184	£1,585,241	£3,923,278
Scenario 6	£1,898,084	£217,774	£1,399,161	£3,515,018
Scenario 7	£1,452,477	£387,149	£1,124,391	£2,964,016
Scenario 8	£1,175,053	£483,188	£956,465	£2,614,699
Scenario 9	£1,831,531	£143,566	£1,509,454	£3,484,551
Scenario 10	£1,580,015	£227,182	£1,345,870	£3,153,067
Scenario 11	£1,242,852	£359,149	£1,128,102	£2,730,103
Scenario 12	£1,018,522	£438,128	£971,924	£2,428,574
Scenario 13	£1,666,528	£153,471	£1,490,022	£3,310,021
Scenario 14	£1,428,968	£231,599	£1,317,754	£2,978,321
Scenario 15	£1,128,107	£352,167	£1,114,044	£2,594,319
Scenario 16	£915,767	£425,174	£954,032	£2,294,973

*NOAC* Novel Oral Anticoagulant treatment, *FU* Follow-up

The policy of shifting more patients towards community services is also beneficial from a cost perspective. This is illustrated by the results for scenarios under which 40% of patients are put on NOAC treatment. The total cost drops from £3,071.872 for scenario 3 (10% community services) to £2,964,016, £2,730,103, and £2,594,316 for scenario 7 (20% community services), scenario 11 (40% community services), and scenario 15 (50% community services), respectively, with the latter (scenario 15) being 44% less compared to the baseline scenario. This total cost reduction is driven by the decrease in the cost of care (standard and NOAC) in the community services setting.

The two policies also lead to total cost reduction if they are both implemented at the same time. For instance, starting from £4,128,567 for scenario 1 (10% both policies), the total cost follows a decreasing slope to £3,515,018, £2,730,103, and £2,294,973 for scenario 6 (20% both policies), scenario 11 (40% both policies), and scenario 16 (50% both policies), respectively. If we compare the latter scenario (scenario 16) to the baseline one, the magnitude of the total cost gain is a significant 50% (from £4,585,941 to £2,294,973).

#### (iv) Value for money

The previous findings focused on the provider side and analysed performance with respect to the operational and cost aspects of delivering care to the patients. However, to achieve a more comprehensive analysis of the best policies to implement, the benefits to the patients need also to be taken into consideration. In this context, a Value for Money (VfM) score is calculated for the 16 scenarios covering the 4 possible interventions from the two policies (NOAC treatment in hospital, NOAC treatment in CSs, Standard treatment in hospital, Standard treatment in CSs) under consideration in this study.

The VfM scores were calculated using the STAR methodology (see Appendix 1). The inputs required to calculate the VfM score include the number of patients receiving the intervention, the total cost of delivering the intervention, patients’ health benefits from the intervention, and the likelihood that the patients will benefit from the intervention. While some inputs such as the number of patients receiving the intervention are calculated from the simulation model for the different scenarios, others, for example, patients’ health benefits are estimated from discussions with clinical staff involved in the care and management of the patients.

The results regarding the VfM score are presented in Table 5 for the 4 possible interventions cited above. The input variables used to calculate the VfM score for each intervention include (i) “Number of patients”, which represents the total number of patients to whom the intervention is applied (calculated from the simulation model for each scenario), (ii) “expected health benefit”, which represents the total health benefits expected to be achieved by the patients to whom the intervention is applied (informed by the expert judgment of clinical staff), and (iii) “total cost”, which represents the cumulative cost of applying the intervention to the relevant patients (calculated from the simulation model for each scenario). The numerical values of these inputs are included in the online supplementary table for all scenarios. It is clear that NOAC-based interventions have a better VfM score than the standard treatment-based ones, and this is the case for every scenario (a higher VfM score indicates better cost-effectiveness of the intervention). For example, for scenario 5, the VfM score under the intervention “NOAC treatment in CSs” is 295.7 compared to a score of 64.6 under the intervention “Standard treatment in CSs”. Under the same scenario, the VfM score for the intervention “NOAC Treatment in hospital” is 103.9 whereas it is 25.0 for the intervention “Standard treatment in hospital”.

**Table 5:** Cost-effectiveness performance (Value for Money score) for the interventions

Scenario	NOAC treatment in CSs	Standard treatment in CSs	NOAC treatment in Hospital	Standard treatment in hospital
Baseline	N/A	N/A	N/A	N/A
Scenario 1	336.4	66.1	111.2	25.0
Scenario 2	251.4	64.7	115.1	25.5
Scenario 3	194.1	68.9	121.1	25.1
Scenario 4	174.2	65.7	119.2	25.2
Scenario 5	295.7	64.6	103.9	25.0
Scenario 6	237.1	64.2	112.7	25.9
Scenario 7	186.0	67.1	121.3	25.1
Scenario 8	176.7	68.3	121.3	25.5
Scenario 9	258.1	66.2	89.0	25.0
Scenario 10	219.2	68.0	106.2	25.4
Scenario 11	189.8	68.5	119.6	24.7
Scenario 12	185.4	69.4	120.1	25.3
Scenario 13	236.5	64.3	86.1	25.2
Scenario 14	207.4	66.9	105.5	25.7
Scenario 15	184.8	67.2	121.1	25.0
Scenario 16	182.5	68.0	121.1	26.3

*NOAC* Novel Oral Anticoagulant treatment. *CSs* Community Services

The other finding is that treating patients in a CSs setting yields always a better VfM in comparison to the hospital setting regardless of the type of treatment. To illustrate, under scenario 5, the VfM score is 295.7 for the intervention “NOAC treatment in CSs” versus 103.9 for the intervention “NOAC treatment in hospital”, and it is 64.6 for the intervention “Standard treatment in CSs” versus 25.0 for the intervention “Standard treatment in hospital”.

**Fig. 6:**
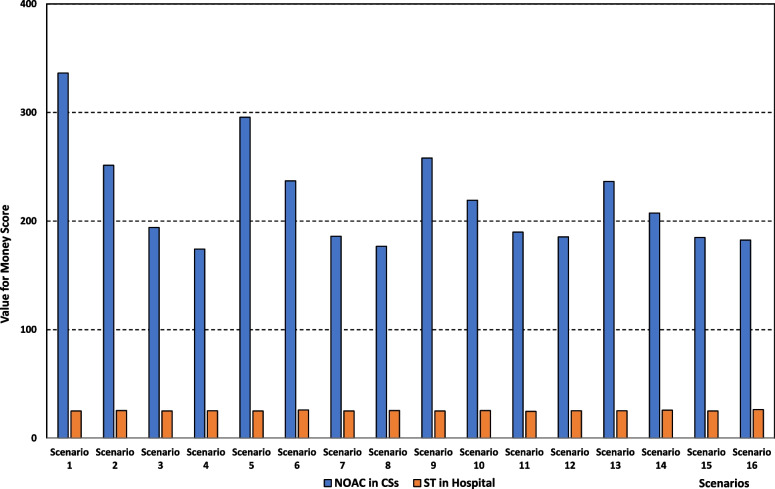
Value for Money scores for treatment with NOAC in community services and for Standard Care in hospital. *NOAC* Novel Oral Anticoagulant treatment, *CSs* Community Services, *ST* Standard Treatment

Comparing the intervention where both policies are implemented (NOAC treatment in CSs) to the one where neither (Standard treatment in hospital) indicates a significant superiority, from a value for money perspective, of the former intervention (See Fig. 6). The VfM score for the intervention “NOAC treatment in CSs” is considerably higher than the intervention “Standard treatment in hospital” and this is the case for all scenarios. For example, under scenario 9, the VfM score is 258.1 for the intervention “NOAC treatment in CSs”, whereas it is a mere 25 for the intervention “Standard treatment in hospital”.

Shifting more patients to the NOAC treatment has opposite effects on the VfM score for those treated with NOAC in CSs and hospital, although the score is always higher in CSs. The score increases in the hospital setting and decreases in the CSs setting (for all fractions of patients shifted to CSs). Assuming that 20% of patients are shifted to CSs, the VfM score for the intervention “NOAC treatment in CSs” decreases from 295.7 under scenario 5 (20% NOAC) to 176.7 under scenario 8 (50% NOAC). Conversely, the VfM score for the intervention “NOAC treatment in hospital” increases from 103.9 under scenario 5 (20% NOAC) to 121.3 under scenario 8 (50% NOAC). It is important to note that the policy of shifting more patients to NOAC has a little impact on the VfM score for the interventions “Standard treatment in CSs” and “Standard treatment in hospital”.

Regarding the effect of moving more patients to CSs, the results indicate a slightly decreasing slope with regard to the VfM score for patients treated with NOAC in both settings (for all fractions of NOAC patients). For example, if 20% of patients are undertaking NOAC treatment, the VfM score decreases from 251.4 and 115.1 under scenario 2 (10% CSs) to 207.4 and 105.5 under scenario 14 (50% CSs) for the interventions “NOAC treatment in CSs” and “NOAC treatment in hospital”, respectively. Similar to the finding in the previous paragraph, the policy of moving more patients to CSs does not influence the VfM score for the interventions “Standard treatment in CSs” and “Standard treatment in hospital”.

## Discussion

This article sits in this context by exploring whether the introduction of the new NOAC treatment and increasing the use of community services as a mode of care delivery for DVT patients can achieve performance improvements from efficiency, resource requirements, costs, and value for money perspectives.

The findings indicate that the new NOAC treatment may potentially provide superior performance compared to the standard treatment both for the provider (NHS) and the patient. It also may reduce the number of visits to hospitals and human resources requirements, freeing much needed capacity. Furthermore, by reducing costs and increasing value for money, NOAC enables the NHS to provide care to DVT patients in a sustainable and cost-effective way, which is important given the financial constraints faced by the organisation.

Increasing the use of community services to care for DVT patients is associated with significant benefits in terms of hospital activity, costs, and value for money. This mode of delivery is, therefore, a promising way, which can help the NHS cope with the increasing demand without significantly inflating costs while offering good value for money benefiting both the patient and the NHS. These performance improvements are achieved regardless of the type of treatment provided to patients but are more significant if coupled with NOAC treatment. This provides evidence that the NHS should firmly integrate this setting as part of its DVT treatment model in the future.

Providing patients with NOAC and treating them in community services reduces significantly hospital activity, human resources requirements, and costs. This has enormous benefits to the NHS as it frees much needed hospital capacity to deal with increased demand. It also contributes to the NHS efforts to recover from the impact of COVID-19 and deal with the enormous backlog of patients waiting for treatment, which grow from 4.43 million patients in February 2020 to 6 million in December 2021 [36].

Shifting more patients to community services comes with some prerequisites. More such services need to be set up to provide capacity and to make it closer to patients, many of whom are elderly people who will benefit from short travel journeys to receive treatment. In addition, investments in hiring, training, and retaining staff are required so that the services are well-resourced from a human capacity perspective. This is a worthwhile investment as, in addition to efficiency, cost, and economic gains, it has been reported that patients feel more comfortable and have less anxiety when treated in these services, enhancing their experience and quality of care [3, 37].

This study provides further evidence regarding the usefulness of simulation techniques such as DES in informing the decision-making process in healthcare. The ability of DES to represent the complexity of patients’ pathways and predict the likely consequences of interventions before their implementation, provides decision-makers with valuable information and evidence for policy-making. This reduces significantly the risks associated with policy selection and prevents costly experimentation, and “doing things and hoping for the best” approach, which is no longer possible given the NHS context and constraints. This significant DES advantage explains its increasing popularity within the healthcare sector [16].

The application of the STAR method to carry out the cost-effectiveness analysis of the policy interventions considered in this study broadens the scope of the analysis beyond the usual focus on operational efficiency and costs. By including health economics performance measures, this study offers a more comprehensive evaluation of interventions covering both the provider and the patient perspectives and enabling a more robust decision-making process. Furthermore, as the STAR method involves participants from the health context, this increases its acceptability and the likelihood of implementing the best interventions identified by the analysis in the real world.

Combining DES, which has the ability of representing the operational aspects of the patients’ pathways and STAR, with its capability of assessing the health economic implications of interventions, provides a comprehensive and robust evidence regarding the most beneficial policies to implement. It offers decision-makers rich information they can rely on to select policies with confidence. In this context, this study provides another example of how successful integration of DES and health economics can lead to better decision-making in the healthcare sector [24, 38].

QALYs are mostly used as a measurement in health economic analysis. However, health scores used in the STAR approach include experts' evidenced opinions considering changes in quality and length of life. Thus, a comprehensive and diverse environment should be set in interviews/workshops to prevent any positive favour toward a new policy/intervention.

Both DES and STAR require interaction with and active participation of stakeholders and staff over the whole study life cycle. This improves validity and trust in the findings, enhances the sense of ownership by participants, and facilitates the implementation process [39]. This helps addressing the long-running problem of mistrust and low implementation of the findings of modelling-based studies in the healthcare sector [17].

## Conclusion

This study investigated whether the introduction of NOAC treatment for patients with DVT and moving patients from the expensive hospital-based treatment to community services will generate better operational and cost performance, and value for money for the NHS. The results indicate that significant gains can be achieved if these interventions are implemented. These are welcomed findings given that the NHS is facing a multitude of challenges and this situation has been compounded by the COVID-19 pandemic.

It is welcoming news that, as part of the long-term strategy (2019/20–2023/24), the NHS has committed to boosting investment in community health services, so that responsive community care is provided for those who are clinically considered to benefit most. This is in line with the policy implications of this study and demonstrates that there are innovative drugs and treatment delivery processes, which can help the NHS deliver care in an efficient and cost-effective way to cope with the rising demand.

The successful combination of DES and health economics in this study gives credit to these methods and provide a template on how healthcare problems can be analysed, and policies and interventions evaluated to improve the decision-making quality in the healthcare sector. This can only be welcomed as healthcare organisations around the world are expected to continue improving their performance and find new and innovative ways to deliver care to cope with the upward trend in demand in a financial and resources constrained environment.

### Supplementary Information

**Additional file 1.**

## Data Availability

Data are available upon reasonable request. All data relevant to the study are included in the article or in supplementary information files.

## Appendix 1: Short description of the STAR method

We adapt the Socio-Technical Allocation of Resources (STAR) approach and integrate this concept into our discrete-event simulation (DES) model to assess the cost-effectiveness (and health benefits) of the variety of treatment options and configurations for Deep Vein Thrombosis (DVT) patients. The STAR approach is modified so that it is fit for our purpose and formulated as follows:

- *Cost* $({c}_{j}$): the cost of the pathway for patients in intervention *j*
- *Population health benefit* $$\left({N}_{j}\times {B}_{j}\right)$$: the number of patients who benefit from the intervention ($${N}_{j}$$) is multiplied by potential benefit ($${B}_{j})$$, expressed as quality (and length) of life. Typically, this is measured using QALY but due to the nature of the STAR approach, the health benefit scores were decided based on the evidence brought by clinical experts considering the potential improvement in quality of life and length of life. DVT specialists were asked to identify the intervention/configuration that provided the highest benefit to patients (scored as 100). Physicians and nurses then scored the remaining interventions relative to this benchmark (scoring from 0 – 100). This scoring makes the comparison of the health benefits of these interventions easier and provides an easy-to-understand graphical comparison.
- *Feasibility* $({p}_{j}$): Probability of success of achieving the assessed benefits. Probability of success means that the intervention (Warfarin or NOAC) can be successfully implemented in practice. Stakeholders were asked to provide a probability of success ranging 0–1 during the interviews/workshops.
- Expected benefit $E\left({v}_{j}\right):$ the expected benefit from intervention *j* calculated as $E\left({v}_{j}\right)= {p}_{j}\left({N}_{j}\times {B}_{j}\right)$. The objective is to Max ${\sum }_{j}E({v}_{j})$.
- Value for money (VfM): ${VfM}_{j}=\left( \frac{E\left({v}_{j}\right)}{{c}_{j}}\right)\times 1000$

Please note that the original formula for value for money (VfM) does not include the multiplication by 1000. The VfM values were multiplied by 1000 to make easier to compare the results. Otherwise, VfM values would be in four decimal points (e.g., 0.025) which will make the comparison difficult.

## Abbreviations

UK: The United Kingdom
NHS: National Health Service
DVT: Deep Vein Thrombosis
NOAC: Novel Oral Anticoagulant
CS: Community Services
DES: Discrete Event Simulation
SD: System Dynamics
ABS: Agent Based Simulation
CEA: Cost-Effectiveness Analysis
NICE: National Institute for Clinical Excellence
STAR: Socio-Technical Allocation of Resources
HIV: Human Immunodeficiency Virus
HIS: Health Information Systems
DST: Decision Support Tool
INR: Anticoagulation clinic
LWMH: Low Weight Molecular Heparin
VfM: Value for Money
PM: Participative Modelling
