# Supplementary material for: A decision support tool with health economic modelling for better management of DVT patients
Source: Health Econ Rev. 2022 Dec 26;12:65. doi: 10.1186/s13561-022-00412-9 (PMC9790817; doi:10.1186/s13561-022-00412-9)
Supplement: Supplementary file 1 — Additional file 1. [file 13561_2022_412_MOESM1_ESM.docx]

Table S1 Input parameters

|  | **Estimate** | **Distribution** | **Data Collection** |
| --- | --- | --- | --- |
| **Demand** | | | |
| Please specify prevalence rate of DVT within your population | 104.6 per 100,0000 population | Poisson | Martinez et al. (2011) |
| Please specify the proportion of patients with Cancer | 12.20% | Multinomial |  |
| Please specify the % of the diagnosed population with RECURRENT DVT | 19% | Multinomial |  |
| Please specify the annual increase for each year (1-5) of patient arrivals | User specified | Bernoulli | Expert opinion |
| **Diagnosis** | | | |
| **What is the percentage of DVT patients arriving from the following sources?** |  |  |  |
| % arriving from the GP Direct  % arriving from Community Care  % arriving from outpatients  % arriving from Accident and Emergency (A&E) | User specified | Multinomial | Expert opinion |
| **What percentage of DVT patients will have a 'Wells Score' of...** |  |  |  |
| have a 'Wells Score' of greater than 2 | 46% | Bernoulli | Wells et al. (2003) |
| have a 'Wells Score' of less than or equal to 2 | 54% |  |  |
| **What percentage of DVT patients will have a...** |  |  |  |
| positive 'D-Dimer' test result | 30% | Bernoulli | Wells et al. (2003) |
| negative 'D-Dimer' test result | 70% |  |  |
| **What % of people will have a…** |  |  |  |
| positive first ultrasound | 24% | Bernoulli | Goodacre et al. (2006) |
| positive repeat ultrasound | 1% |  |  |
| **What percentage of DVT patients will have…** |  |  |  |
| Unprovoked | 44.80% | Multinomial | Martinez et al. (2011) |
| Provoked | 36.20% |  |  |
| Recurrent | 19.0% |  |  |
| **Treatment** | | | |
| **What percentage of patients will receive the following options?** |  |  |  |
| Standard of Care (LMWH + Warfarin) | User specified | Bernoulli | Expert opinion |
| New treatment |  |  |  |
| **Please define the % of patients and length of treatment** |  |  |  |
| For **Recurrent group** | 3 months: 3%  6 months: 42%  12 months: 55% | Multinomial | Bauersachs et al. (2010) |
| For **Provoked group** | 3 months: 22%  6 months: 63%  12 months: 15% | Multinomial |  |
| For **Unprovoked group** | 3 months: 6%  6 months: 63%  12 months: 31% | Multinomial |  |
| **Please indicate the length of treatment for cancer patients** | 6 months: 100% | Fixed | Noble et al. (2008) |
| **Please indicate the number of follow up visits required** |  |  |  |
| **Warfarin** | 3 months: 9  6 months: 14  12 months: 24 | Poisson | Winter et al. (2005), Rose et al. (2011), Keeling et al.  (2011) |
| **LMWH** | 8 days: 8 | Fixed | (Scottish Intercollegiate  Guidelines Network, 2010) |
| **New treatment** | 3 months: 2  6 months: 2  12 months: 2 | Poisson | New treatment  Prescribing Information (PI) |
| **On average how much staff time does it take to conduct a FIRST visit to the DVT clinic** | User specified | Average | Expert opinion |
| **Please indicate the % of time each type of staff is responsible** |  |  |  |
| Haematologist | User specified | Multinomial | Expert opinion |
| Nurse | User specified | Multinomial | Expert opinion |
| Radiologist | User specified | Multinomial | Expert Opinion |
| **On average how much staff time does it take to conduct a FOLLOW-UP visit to the DVT Clinic** | User specified | Average | Expert Opinion |
| **Please indicate the percentage of time each type of staff is responsible** |  |  |  |
| Haematologist | User specified | Multinomial | Expert Opinion |
| Nurse | User specified | Multinomial | Expert Opinion |
| Radiologist | User specified | Multinomial | Expert Opinion |
| **Costing** | | | |
| Cost of FIRST Visit to Haematology/DVT clinic | £247 | Fixed | Payment by Result (2013) |
| Cost of FOLLOW-UP Visit to Haematology/DVT clinic | £113 | Fixed | Payment by Result (2013) |
| Cost of FIRST visit community DVT clinic | User specified | Fixed | Expert opinion |
| Cost of FOLLOW-UP visit community DVT clinic | User specified | Fixed | Expert opinion |
| New treatment price per tablet | £2.10 | Fixed | (MIMS) |
| LMWH (NON-CANCER) cost per day | £9.77 | Fixed | (MIMS), assuming an average patient weight of 80kg |
| LMWH (CANCER) cost per day month 1 | £8.47 | Fixed | (MIMS) assuming an average weight of a patient 69-82Kg |
| Warfarin cost per day | £0.07 | Fixed | (MIMS) |
| **Salary - Hourly Cost** | | | |
| Haematologist | £139 | Fixed | PSSRU (2013) |
| Nurse | £123 | Fixed | PSSRU (2013) |
| Radiologist | £139 | Fixed | PSSRU (2013) |
| **Number of resources** | | | |
| Haematologist | User specified | Fixed | Expert opinion |
| Nurse | User specified | Fixed | Expert opinion |
| Radiologist | User specified | Fixed | Expert opinion |

**Notes:** DVT: deep vein thrombosis; GP: General Practice; LMWH: Low molecular weight heparin

**References**

Bauersachs R, Berkowitz SD, Brenner B, Buller HR, Decousus H and Gallus AS (2010). Oral Rivaroxaban for Symptomatic Venous Thromboembolism. *The New England Journal of Medicine* **363**: 2499-2510.

Goodacre S, Sampson F, Stevenson M, Wailoo A, Sutton A, Thomas S et al. (2006). Measurement of the clinical and cost-effectiveness of non-invasive diagnostic testing strategies for deep vein thrombosis. *Health Technology Assessment* **10**: 1-168.

Keeling D, Baglin T, Tait C, Watson H, Perry D, Baglin C, et al. (2011). Guidelines on oral anticoagulation with warfarin - fourth edition. *British Journal of Haematology* **154**: 311-324.

Martinez C, Rietbrock S, Bamber L and Cohen AT (2011). Incidence of venous thromboembolism (VTE) in the general population - VTE Epidemiology Group study. XXIII Conference of The International Society on Thrombosis and Haematosis (ISTH).

MIMS. (n.d.). Prescription drug database. Retrieved March 1, 2014, from http://www.mims.co.uk/

Noble SI., Shelley MD, Coles B, Williams SM, Wilcock A and Johnson MJ (2008). Management of venous thromboembolism in patients with advanced cancer: a systematic review and meta-analysis. *The Lancet Oncology* **9**: 577-584.

Payment by Result. (2013). Payment by Results in the NHS: tariff for 2012 to 2013. Retrieved June 23, 2013, from https://www.gov.uk/government/publications/confirmation-ofpayment-by-results-pbr-arrangements-for-2012-13

PSSRU. (2013). Unit Costs of Health and Social Care. Retrieved June 1, 2013, from http://www.pssru.ac.uk/project-pages/unit-costs/2013/#sections

Rose P, James R., Chapman O, and Marshall S (2011). A real world evaluation to describe the characteristics, outcomes and resource use associated with patients being managed by a secondary care based anticoagulation service. *Value in Health* **14**: A387-A388.

Scottish Intercollegiate Guidelines Network. (2010). Prevention and management of venous thromboembolism. Retrieved March 1, 2014, from http://www.sign.ac.uk/pdf/sign122.pdf Sterman J D (2000). Business dynamics. Systems thinking and modeling for a complex world. Mc-Graw Hill: Singapore.

Wells PS, Anderson DR, Rodger M, Forgie M, Kearon C, Dreyer J, et al. (2003). Evaluation of D-Dimer in the Diagnosis of Suspected Deep-Vein Thrombosis. *The New England Journal of Medicine* **349**: 1227-1235.

Winter M, Keeling D, Sharpen F, Cohen H and Vallance P (2005). Procedures for the outpatient management of patients with deep vein thrombosis. *Clinical and Laboratory Haematology* **27**: 61-66.
